# Pneumomediastinum as initial presentation of paralytic rabies: A case report

**DOI:** 10.1186/1471-2334-5-92

**Published:** 2005-10-25

**Authors:** Pongtorn Kietdumrongwong, Thiravat Hemachudha

**Affiliations:** 1 Department of Emergency Medicine, King Chulalongkorn Hospital, Bangkok, Thailand; 2 Department of Medicine, Chulalongkorn University Hospital, Bangkok, Thailand

## Abstract

**Background:**

Rabies is readily diagnosed when it presents as the classic furious form. Paralytic and atypical forms can pose significant problems in diagnosis. Catastrophic incidents included 7 organ transplant recipients who died of rabies recently in United States and Germany. Although rabies remains top in the lists of differential diagnosis of encephalitis in rabies endemic area, its complication may divert physicians from making a relevant management. We encountered an unusual case of paralytic rabies who presented with spontaneous pneumomediastinum.

**Case Presentation:**

A young male presented with fever and dysphagia. There was a history of fluctuating consciousness and aerophobia but they were absent or could not be demonstrated at the time of admission. He exhibited subcutaneous chest wall emphysema and was found to have pneumomediastinum which resulted in surgical intervention. He developed paralysis followed by seizures during postoperative period. Diagnosis was confirmed by demonstration of rabies RNA in saliva during the preterminal phase and by the autopsy. Over 200 hospital staff subsequently received rabies postexposure prophylaxis.

**Conclusion:**

Spontaneous pneumomediastinum can be a rare complication of rabies. It may lead clinicians to perform inappropriate treatment, particularly when phobic spasms are not present and agitation is not prominent. High level of awareness of rabies in any patient with confusion albeit subtle or with any obscure neurological presentations such as difficulty swallowing with no identifiable causes must be borne in mind.

## Background

Rabies is an acute viral encephalomyelitis which is virtually 100% fatal. The disease is prevalent in developing countries where it is underreported. Although rabies incidence in humans significantly declined in Thailand from almost 200 to 20 cases annually during the past two decade, more than 400,000 persons required rabies postexposure prophylaxis (PEP) in 2003. This is more than 4 times as many as in 1991 and may explain the lower prevalence in human disease [1]. Furthermore, the percentage of animal brain samples that were confirmed infected with rabies during the 10 year period remained unchanged, within the range of 23–30%. This documents that the main natural vector for rabies in Thailand (the dog) is a remaining threat. Clinical presentations in humans can be categorized as classic (furious and paralytic) and non-classic rabies [2,3]. The latter is almost always associated with bat and some dog rabies variants. Paralytic and non-classic forms are extremely difficult to diagnose. Missing the diagnosis was tragically documented in the United States and Germany where 7 organ transplant recipients died from this disease [4] [ 02/17/2005]. One donor was reportedly bitten by a bat, while the other had a recent travel history to India. Also, several Thai patients who died of rabies had undergone plasma exchanges because of a misdiagnosis of Guillain Barre' syndrome (GBS) [2,5]. Failure to correctly diagnose and manage rabies can be due to unusual clinical manifestations and by distraction of the clinicians by unusual complications [3]. We described a paralytic rabies patient who had dysphagia and pneumomediastinum (ie free air or gas contained within the thoracic cavity from the escape of air into the mediastinal tissues, usually from rupture of interstitial emphysema or pulmonary bleb and also can be found in association with severe asthma, vomiting, excessive coughing or shouting) as initial presentations.

## Case presentation

An 18-year old male from Myanmar was brought to the emergency department because of difficulty in swallowing and alteration of consciousness. He had been in good health and had arrived Bangkok 6 days earlier. Three days prior to admission, he developed fever and difficulty in swallowing. He also had pruritus of his right leg and buttock which resulted in extensive excoriation. He had became intermittently confused and refused to eat or drink. There were episodes of agitation and he was reported to have complained of shortness of breath incited by fanning.

One day prior to entry, he was unable to swallow his saliva. His mental status alternated between normal periods and confusion. He gave a history of dog bite 10 years previously but no post exposure vaccination was administered and he denied any recent animal bites or contact with bats.

**Figure 1:**
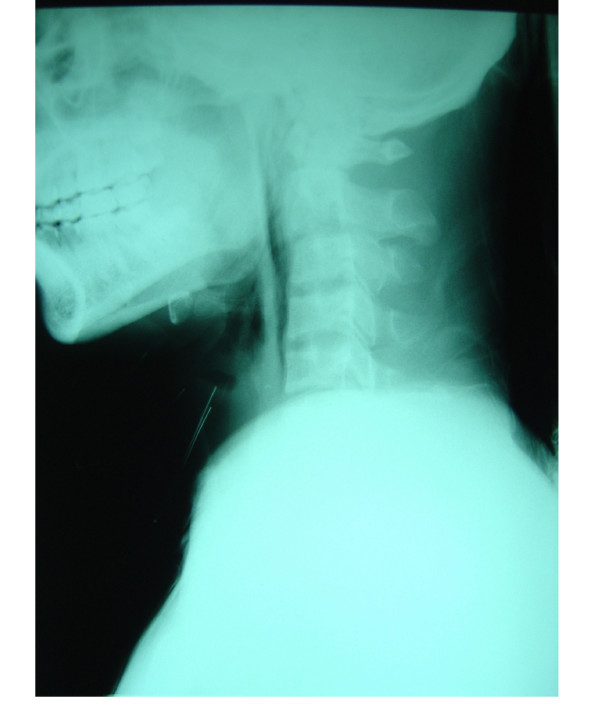
Lateral neck X-Ray showed air in retropharyngeal space.

**Figure 2:**
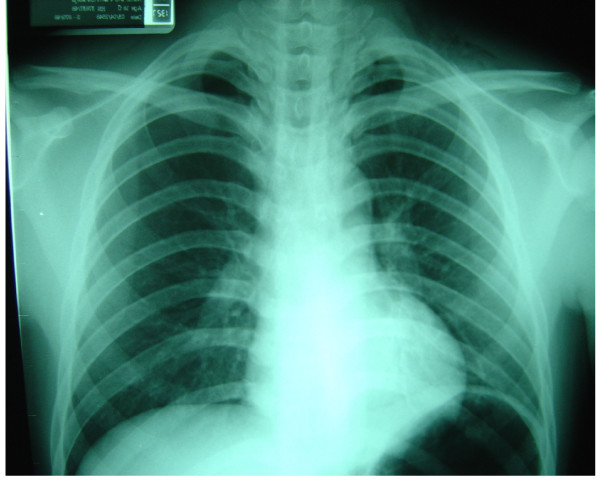
Chest X-Ray showed pneumomediastinum.

On admission, he was conscious and well co-operative. He complained of a severely sore throat. He insisted on sitting and continuously spitting out saliva. His body temperature was 39.1°C, blood pressure 130/70 mmHg, heart rate 120 and respiration 30 per minute. There was crepitus on the left side of his neck without signs of inflammation. There were no focal neurological signs. His palatopharyngeal muscle functions were intact but the gag reflex was hyperactive. Aero- and hydrophobic spasms could not be induced. Indirect laryngoscopic examination showed no abnormality. Laboratory studies revealed a leucocytosis of 28,400 with 89.6% of neutrophils. His serum amylase level was slightly elevated (443 U/L, normal range 28–100 U/L). The computerized tomography (CT) scan of the brain was unremarkable. Lumbar puncture revealed an acellular cerebrospinal fluid (CSF) and normal protein and sugar levels. Lateral neck radiographs showed air in retropharyngeal space (Figure 1) and a chest x-ray performed at the same time showed evidence of pneumomediastinum (Figure 2). A liquid barium esophagogram failed to show any point of leakage. Surgical consultation was obtained for exploration. Between admission and operation, his mental state was clear and he showed no signs of aggression and the differential diagnosis of possible rabies was disregarded.

Operative findings were unremarkable. There was no evidence of perforation in upper esophagus and oropharynx. Retropharyngeal area was normal without any accumulation of fluid or purulent discharge.

He required assisted ventilation postoperatively but was able to move limbs voluntarily. He remained rational and cooperative. His hypernatremia (Na 157 mOsm/L) was rapidly corrected to140 mOsm/L within the following 11 hours. On the second postoperative day, he developed brief generalized tonic- clonic seizures. Intravenous phenytoin was initiated. Serial seizures developed on day 6. Repeated lumbar puncture and CT scan were unremarkable. He was conscious but failed to communicate. Paralysis of all limbs with absence of deep tendon reflexes was noted on the same day. He became comatose on day 9. Paralytic rabies was then suspected and rabies virus RNA was demonstrable in saliva but not in the urine by nucleic acid sequence based amplification technique (3). The patient died after 12 days of hospitalization (15 days after first clinical onset). Autopsy findings showed wide spread Negri bodies through out the whole neural axis.

## Discussion

This case demonstrates how rabies can escape diagnosis or it is relegated to a secondary position in the differential diagnosis even in a rabies experienced medical center. Most textbooks emphasize the manifestations of furious rabies in humans. Phobic spasms, in the form of aero- and hydrophobia, if present, are pathognomonic of rabies. However, these signs may be absent or not present at all time [2,3]. Difficulty in swallowing usually accompanies phobic spasms. Physical examination only yields a hyperactive gag reflex without any sensory changes at the palate or posterior pharyngeal wall. Other cardinal manifestations, such as fluctuating consciousness and autonomic stimulation signs, also appear periodically or may be absent. They are seen in only half of paralytic rabies patients and are usually not prominent. Once coma develops, phobic spasms are replaced by spontaneously occurred inspiratory spasms which may appear only once or twice during five minutes of observation. They can be difficult to detect in cases of paralytic rabies due to severe weakness of neck and accessory respiratory muscles and the diaphragm.

The patient was admitted to a tertiary care hospital in a canine rabies endemic region. Yet, despite a clear history of fluctuating consciousness and fear of wind or air stream exposure as well as difficulty swallowing without an obvious cause, specialists (such as, internists, otolaryngologists, neurologists and surgeons) ignored such history and were impressed by the dramatic clinical signs of subcutaneous emphysema and pneumomediastinum. Lacking a history of rabies exposure is not uncommon in both furious and paralytic rabies patients. Approximately 10% of rabies patients in rabies endemic areas did not report any exposure, although genetic analysis of rabies virus showed an association with dog rabies variant [6,7]. This may be explained by common minor exposures which are being ignored. This is even more common where bat rabies variants are involved [8,9]. Most deaths occurred because individuals were unaware due to the trivial nature of the wound inflicted by bats and, therefore, they did not seek effective treatment[10]. Some bat rabies variants possess an unique cellular tropism that they can replicate more effectively in the dermis or skin epithelial cells than within muscle cells [11].

Local neuropathic pain, in the form of burning, itching or pruritus, can be found in 30% of dog associated cases and 80% of bat associated cases [12]. This local prodrome was present in this case as pruritus and pain at his right leg and buttock. This was severe to cause extensive excoriation. Dorsal root ganglioneuronitis had been shown to be responsible for such reactions [5].

The findings of air in retropharyngeal space and pneumomediastinum distracted attention from the other history and other symptoms that were suggestive of rabies. We found only one previous report of spontaneous pneumomediastinum in a rabies patient [13], but there was another report of post-mortem finding of ruptured esophagus [14]. The mechanism of spontaneous pneumomediastinum and ruptured esophagus is unknown but may well be due to the violent spasms. Hypernatremia and seizures, well recognized complications are less common in dog associated cases particularly the paralytic type [15,16]. They appeared during the postoperative period in this case. Seizures were suspected to be the consequence of rapid correction of hypernatremia. Percussion myoedema, which can be elicited by tapping on the deltoid region with a tendon hammer and results in swelling of the tissue for a few seconds, was not examined for in this case [17]. Lumbar puncture was performed twice on initial admission and during the time he was comatose. Normal results are typical following rabies infection and may impact on clinical judgment. This was also true in CT scan. [18]. Unlike Japanese encephalitis or its counterparts in a family of *Flaviviridae*, CT scans are often normal in rabies.

Result of nucleic acid sequence based amplification and the autopsy findings confirmed rabies. Positive result on the saliva but not in the urine was not surprising [19-21]. Rabies virus is excreted intermittently and not simultaneously in all types of secretions (for example, saliva, CSF, urine). Sequencing analysis of the nucleoprotein gene showed that the virus could be categorized in a certain clade of dog rabies variant circulating in Thailand (data not shown).

Over 200 hospital staff received rabies PEP. Later analysis made it doubtful that they all had a potential rabies exposure according to the WHO recommendations [22, 23, 24]. Such anxiety reactions have been observed commonly after hospital deaths of rabies patients. We use an alternative accelerated pre-exposure schedule using an intradermal (ID) regimen for nursing and other staff, which might be exposed. It consists of two ID injections at 2 sites (0.1 ml tissue culture vaccine per site) at both deltoids on days 0, 3 and 7. However, this regimen is not currently recommended by WHO.

In summary, this is a case of classic paralytic rabies. Absence of physical signs and symptoms does not exclude a rabies diagnosis. Greater effort to make an early diagnosis, in this particular case, would have prevented additional suffering for the patient who should have been given palliative care instead of unnecessary surgical intervention. Detailed history and analysis of the whole clinical scenario would avoid such disastrous event and consequence. Pre-exposure vaccination for health workers at risk of exposure should be recommended.

## Competing interests

The author(s) declare that they have no competing interests.

## Authors' contributions

Dr. Pongtorn Kietdumrongwong took care of the patient and helped coordinating with physicians from various departments and wrote the paper. Dr. Thiravat Hemachudha was consulted during the pre-terminal phase in the intensive care unit and analyzed clinical specimens for rabies RNA detection and wrote the paper.

## Pre-publication history

The pre-publication history for this paper can be accessed here:


